# An Unusual Case of Severe Cystic Lung Disease: A Case Report and Review of the Literature

**DOI:** 10.7759/cureus.23442

**Published:** 2022-03-24

**Authors:** Sofia Lakhdar, Deesha Shah, Laura M Guzman Perez, Christina Sneed, Theo Trandafirescu

**Affiliations:** 1 Internal Medicine, Queens Hospital Center, New York, USA; 2 Internal Medicine, Icahn School of Medicine at Mount Sinai, Queens Hospital Center, New York, USA; 3 Medicine, Icahn School of Medicine at Mount Sinai, Queens Hospital Center, New York, USA

**Keywords:** rare lung disease, lymphangioleiomyomatosis, birt-hogg-dube syndrome, pulmonary langerhans cell histiocytosis, lung cystic disease, amyloid cystic lung disease

## Abstract

Cystic lung diseases are a heterogeneous group of disorders with varying presentations and pathophysiology. They present as air-filled lung cysts that are prone to rupture and result in spontaneous pneumothoraxes. While pulmonary cysts are not uncommon, cysts presenting later in life with unclear etiology are rare and result in both a diagnostic and therapeutic challenge. In this report, we present a case of an 82-year-old female presenting with shortness of breath and hemoptysis. Computed tomography (CT) angiogram showed multiple pulmonary cysts with one of the cysts containing an air-fluid level suspicious of superimposed infection. Pulmonary cysts are characteristic of different diseases that include but are not limited to Langerhans cell histiocytosis (LCH), lymphangioleiomyomatosis (LAM), and Birt-Hogg-Dube (BHD) syndrome. The differential diagnosis of cystic lung disease over the years has become more complex. Clinical context and radiological findings are essential for diagnosis.

## Introduction

Cystic lung diseases are a heterogeneous group of disorders with varying presentations and pathophysiology. Pulmonary cysts are characteristic of different diseases that include but are not limited to Langerhans cell histiocytosis (LCH), lymphangioleiomyomatosis (LAM), and Birt-Hogg-Dube (BHD) syndrome. The differential diagnosis of cystic lung disease over the years has become more complex [1]. Other rare causes of cystic lung disease include follicular bronchiolitis, metastatic disease such as sarcoma, and other hereditary diseases such as Marfan’s syndrome, Ehlers-Danlos syndrome, and Proteus syndrome. Pulmonary cysts need to be differentiated from other conditions that may mimic similar features such as emphysema, cavity, bulla, bleb, pneumatocele, and honeycombing [2]. This is done by evaluating the thickness of the wall, size of gas-filled space, and anatomic location of the cysts [3].

## Case presentation

An 82-year-old female with a medical history of chronic obstructive pulmonary disease (COPD), heart failure with reduced ejection fraction (HFrEF), coronary artery disease (CAD) with percutaneous stent placement six years ago, STEMI two years ago, chronic kidney disease stage 3, pulmonary embolus, deep venous thrombosis, endovascular repair of abdominal aortic aneurysm (AAA), Grave’s disease, and hyperparathyroidism presented to the emergency department for productive cough and blood-tinged sputum. The patient reported a productive cough with whitish phlegm for one week with occasional episodes of blood-tinged sputum. The patient endorsed dyspnea on exertion that worsened in the last six months. Notably, she denied any fever, chills, nausea, vomiting, hematemesis, abdominal pain, weight loss, and changes in bowel or urinary habits. The patient smoked for 45 years and quit around 22 years ago. The patient denied any travel outside the United States. Family history was significant for diabetes mellitus. Her laboratory findings are shown in Table 1.

**Table 1:** Laboratory findings

Laboratory parameter	Result	Reference
White blood cell count	4.11 K/uL	3.80-10.50 K/uL
Hemoglobin	14.2 g/dL	11.5-15.5 g/dL
Hematocrit	44.7%	34.5%-45%
Platelet	180 K/uL	150-400 K/uL
Eosinophil	1.9%	0%-6%
Creatine	1.50 mg/dL	0.50-1.20 mg/dL
Alpha-1 antitrypsin	145 mg/dL	90-200 mg/dL
Fungitell	33 pg/mL	<80 pg/mL
Antinuclear antibody (ANA)	Negative	<1:80
Anti-DNA antibody (DS)	<12 IU/mL	≤29 IU/mL
Rheumatoid factor	<10 IU/mL	0-13 IU/mL
Anti-neutrophilic cytoplasmic antibody	Negative	Negative
SCL70 AB	0.2	≤0.9
Estradiol	16 pg/mL	<5-138 pg/mL (postmenopause)
VEGF	23 pg/mL	0-115 pg/mL

**Figure 1:**
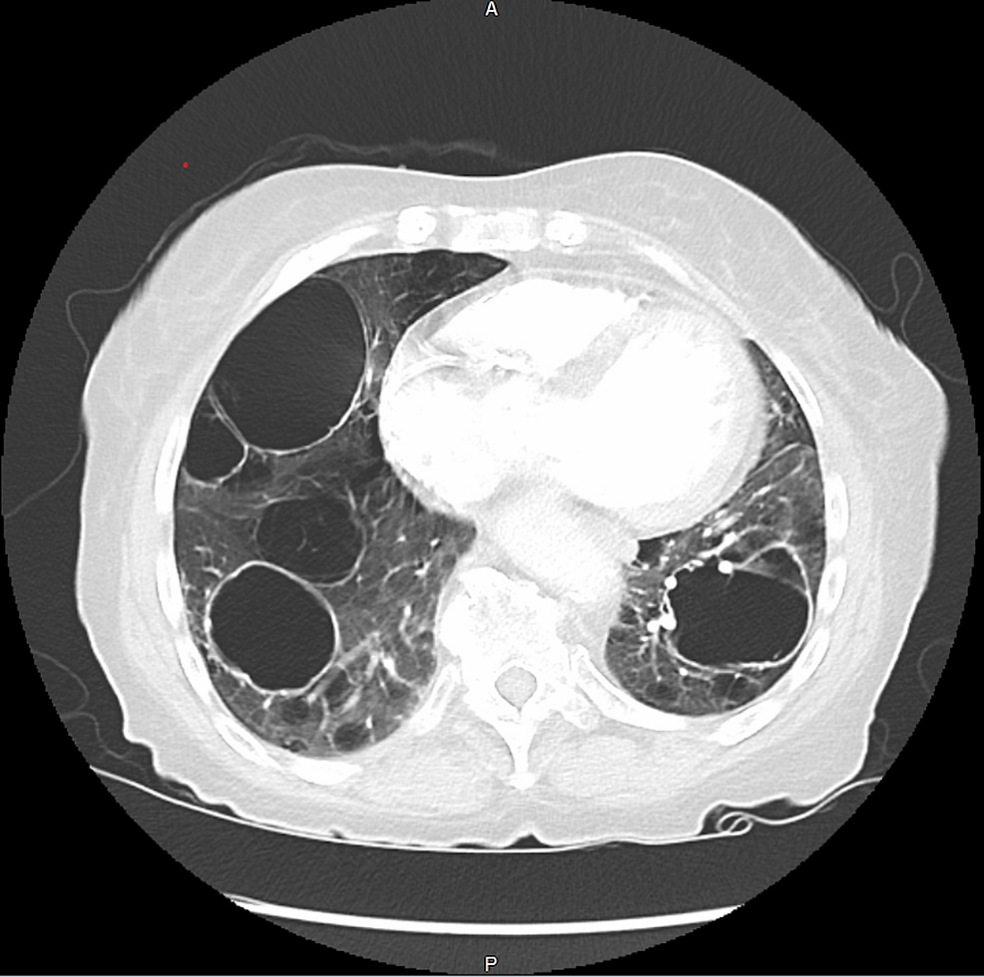
Axial view of the chest CT demonstrating multiple lung cysts

**Figure 2:**
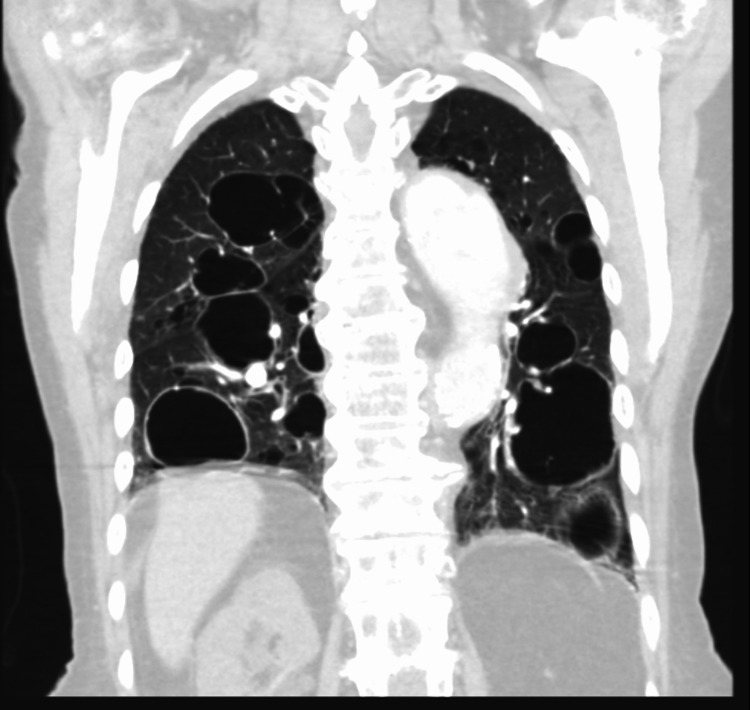
Coronal view of the chest CT demonstrating bilateral cysts

Computed tomography (CT) angiogram showed multiple pulmonary cysts (Figures 1, 2) with one of the cysts containing an air-fluid level suspicious of superimposed infection. Initial workup for cystic lung diseases was performed. Serology was obtained to evaluate for possible underlying rheumatological, hematological, and genetic conditions, which all were found to be inconclusive. Serum IgE was also obtained and was negative. Serum VEGF-D was not obtained. Imaging was not found to be suggestive of centrilobular emphysema, and the appearance of the cysts was not consistent with emphysematous bullae. Although invasive aspergillus was part of the differential, the imaging was not consistent with mycetoma and thus was also unlikely in this patient. The cysts appeared to be homogeneous. Further diagnostic testing with bronchoscopy and tissue biopsy would have increased the risk of possible pneumothorax and therefore were not performed. The patient was managed conservatively for community-acquired pneumonia with antibiotics and bronchodilators and was discharged after symptoms improved.

## Discussion

Pulmonary cysts are characteristic of different diseases, and the differential diagnosis over the years has become more complex [1]. They can be classified based on the underlying pathophysiologic mechanisms: congenital, genetic, infectious, inflammatory, lymphoproliferative, neoplastic, and smoking-related [4]. The patient presented in the case had a very indolent clinical course, and the diagnosis was difficult to achieve. Here, we discuss and highlight the important findings of cystic lung diseases. LAM is a rare disease that affects women of childbearing age. It may occur sporadically or be associated with a tuberous sclerosis complex (TSC) [1]. Histologically, LAM is characterized by the proliferation of neoplastic smooth muscle cells, known as LAM cells, in the lung parenchyma [5]. Cysts are commonly round, mostly 2 mm to 2 cm in size, and thin-walled [1]. Extrapulmonary findings include renal angiomyolipoma, chylous ascites, and stigmata of tuberous sclerosis. Typical cystic findings indicative of LAM can be identified on CT, without invasive procedures such as lung biopsy, if more than one of the following are present: TSC, angiomyolipoma, chylothorax, lymphatic involvement, or elevated serum VEGF-D [6]. On the other hand, pulmonary Langerhans cell histiocytosis (LCH) is a rare lung disease characterized by the accumulation of Langerhans cells in small airways and is usually seen in young adult smokers [7] between the age of 20 and 40 years with no gender predilection [8]. About 90%-100% of those diagnosed with LCH have a current or prior history of smoking [9,10]. CT scan of the chest shows nonspecific nodular/reticulonodular opacities. High-resolution CT (HRCT) of the chest shows the characteristic findings of pulmonary nodules and/or cysts predominately in the upper and middle lobes. Cysts are also bizarre in shape as compared to LAM or BHD [2]. Pulmonary LCH is known to involve predominantly the upper and middle lobes of the lungs while sparing the bases and is characterized by the formation of pulmonary nodules in the early stages and thin-walled pulmonary cysts in the later stages. In the end stages of the disease, the cysts are thought to coalesce together and form large thin-walled pulmonary cysts [10]. Although the CT findings and symptomatology can point toward the disease, confirmation of the diagnosis is usually needed and done via lung biopsy and/or BAL.

Lymphoid interstitial pneumonia (LIP) is an extremely rare and benign lymphoproliferative disorder and is commonly seen associated with patients with connective tissue disorders, such as Sjogren’s syndrome (SS), systemic lupus erythematosus, and rheumatoid arthritis, or immune-deficiency states [11]. It typically follows a lymphatic distribution [12]. LIP usually affects females between the age of 40 and 70 years [12]. On HRCT, the cysts in LID are randomly distributed, have an internal structure, measure <30 mm in diameter, and are typically fewer than in LAM [2]. BHD syndrome is a rare inheritable multisystem condition most commonly affecting the skin, lungs, and kidneys. It is an autosomal dominant syndrome characterized by cutaneous fibrofolliculomas, multiple lung cysts, spontaneous pneumothorax, and renal cancer. However, this condition is heterogeneous and does not need to affect multiple systems for diagnosis. Gender preference appears to be equal with no significant risk in male versus female populations. Symptoms have been reported in as young as the age of seven but more commonly present in patients in their fourth or fifth decade of life [2]. On HRCT, the cysts in BHD syndrome are multiple, thin-walled, round or lentiform, and well-defined, and size ranges from 2 mm to 78 mm [4]. The lung volume is commonly preserved or increased, but no other significant pulmonary involvement has been described [4]. The cysts sometimes have an intracystic septum, with an “alveoli within an alveolus” appearance [4]. Patients with BHD may also present with dermatologic lesions. Renal tumors after age 40 are also common. However, renal pathology in these patients can be diverse and present as simple as a renal cyst in some patients [13].

Amyloidosis of the lung tends to occur in the sixth decade of life [14]. Amyloidosis results from a misfolded protein that cannot be degraded by cellular enzymes or ubiquitination. This misfolded protein accumulates and can damage tissues. The accumulation of amyloid deposits is more commonly seen in the kidneys, heart, central nervous system, and gastrointestinal tract, but many cases have been also reported in the lungs. Confirmation is by tissue biopsy demonstrating amorphous material that stains positively with Congo red [1]. Amyloid deposition in the respiratory tract can result in tracheobronchial disease, parenchymal cysts/nodules, and infiltrates, pleural disease, and lymphadenopathy. Isolated pulmonary amyloidosis has been noted to have a benign clinical course with a good prognosis, but pulmonary amyloidosis associated with systemic disease is associated with a poor long-term outcome after diagnosis [15]. Pulmonary amyloidosis cysts are associated with collagen vascular disease, most commonly Sjogren’s syndrome (SS) [15]. Pulmonary mucosa-associated lymphoid tissue lymphoma (MALToma) was also associated with cystic lung disease due to amyloidosis [16]. Cystic disease of the lung due to amyloidosis does not appear to be associated with a smoking history. Radiologic findings on chest CT in patients with pulmonary amyloidosis cysts present as multiple thin-walled cysts that are round or lobulated in shape and small to moderate in size and are usually more peripheral in location [16]. CT findings may show interlobular septal thickening, honeycombing, ground-glass opacities, circumferential thickening of the tracheal wall, and lymphadenopathy [16].

## Conclusions

A variety of pathologies can present as lung cysts and cystic lung disease. In patients who are found to have severe cystic lung disease, it is difficult to establish a clear diagnosis given the timing of presentation and overlapping imaging findings.
